# High-Phytate Diets Increase Amyloid β Deposition and Apoptotic Neuronal Cell Death in a Rat Model

**DOI:** 10.3390/nu13124370

**Published:** 2021-12-06

**Authors:** Hyo-Jung Kim, Yun-Shin Jung, Yun-Jae Jung, Ok-Hee Kim, Byung-Chul Oh

**Affiliations:** 1 Department of Physiology, Lee Gil Ya Cancer and Diabetes Institute, Gachon University College of Medicine, Incheon 406-840, Korea; gywjd1990@naver.com (H.-J.K.); hyunshinm@gmail.com (Y.-S.J.); 2 Department of Microbiology, Lee Gil Ya Cancer and Diabetes Institute, Gachon University College of Medicine, Incheon 406-840, Korea; yjjung@gachon.ac.kr

**Keywords:** high-phytate diet, Ca^2+^ and Pi dyshomeostasis, amyloid-β, APP processing, APP, β-secretases (Bace1), retinoid X receptor

## Abstract

Amyloid-β (Aβ) accumulation in the hippocampus is an essential event in the pathogenesis of Alzheimer’s disease. Insoluble Aβ is formed through the sequential proteolytic hydrolysis of the Aβ precursor protein, which is cleaved by proteolytic secretases. However, the pathophysiological mechanisms of Aβ accumulation remain elusive. Here, we report that rats fed high-phytate diets showed Aβ accumulation and increased apoptotic neuronal cell death in the hippocampus through the activation of the amyloidogenic pathway in the hippocampus. Immunoblotting and immunohistochemical analyses confirmed that the overexpression of BACE1 β-secretase, a critical enzyme for Aβ generation, exacerbated the hippocampal Aβ accumulation in rats fed high-phytate diets. Moreover, we identified that parathyroid hormone, a physiological hormone responding to the phytate-mediated dysregulation of calcium and phosphate homeostasis, plays an essential role in the transcriptional activation of the Aβ precursor protein and BACE1 through the vitamin D receptor and retinoid X receptor axis. Thus, our findings suggest that phytate-mediated dysregulation of calcium and phosphate is a substantial risk factor for elevated Aβ accumulation and apoptotic neuronal cell death in rats.

## 1. Introduction

Amyloid β is derived from the proteolytic cleavage of the amyloid precursor protein (APP) by APP processing enzymes [1,2], including β-secretase (BACE1) and γ-secretase (PSEN1), thereby generating either Aβ_40_ or Aβ_42_ insoluble peptide [2,3]. The accumulation of insoluble Aβ has been suggested to damage structural and functional brain networks, thus resulting in cognitive impairment [4]. Furthermore, Alzheimer’s disease (AD) can result from autosomal dominant mutations in the *app* gene, or *psen1* and *psen2* genes, which have been found to increase Aβ production, thereby promoting Aβ aggregation and deposition [5]. In late-onset sporadic AD, the accumulation of Aβ in the brain has been attributed to defective Aβ clearance [6], as well as elevated expression and increased enzymatic activity of BACE1 [6]. However, the dietary conditions that facilitate the overexpression of APP and APP processing enzymes in the hippocampus are largely unknown. 

Dietary phytate is the major form of dietary phosphorus in cereals and legumes, accounting for as much as 80% of total phosphate [7,8]. Six phosphate groups of phytates tightly bind essential minerals, such as Ca^2+^, Mg^2+^, Fe^2+^, and Zn^2+^, thus forming indigestible and unabsorbable mineral-phytate salts [8,9] and exacerbating mineral deficiency in humans and other monogastric animals lacking the digestive enzyme phytase, which releases phosphate in the gut [9]. Thus, phytate is considered a negligible source of excessive intestinal phosphate overload [10]. However, we previously showed that phytate dysregulates the homeostasis of both Ca^2+^ and phosphate by decreasing Ca^2+^ absorption while increasing intestinal phosphate overload [11], depending on the dietary Ca^2+^ content. Thus, high-phytate, low-Ca^2+^ diets are a significant risk factor for phosphate overloading and renal phosphate wasting, which are associated with elevated risks of hyperparathyroidism, nephrocalcinosis, vitamin D deficiency, and bone loss in rats [11]. In contrast, diets with high phytate and high Ca^2+^ contents have been found to ameliorate several phytate-mediated pathogenic defects by forming indigestible calcium-phytate [11]. However, despite the harmful effects of dietary phytate on Ca^2+^ and phosphate homeostasis, the effects of dietary phytate on the APP processing enzymes and insoluble Aβ deposition in the hippocampus have not been investigated.

The dysregulation of Ca^2+^ and Pi homeostasis affects various physiological processes in multiple organs, including the bone, liver, muscles, kidney, and brain, thereby often leading to severe illnesses such as chronic kidney disease [12,13], cardiovascular disease [14,15], vascular calcification [16,17], insulin resistance [14], and AD [18,19,20,21]. The dysregulation of intracellular calcium signaling has been implicated in the pathogenesis of AD [22]. Increased intracellular calcium elicits the neuropathology of AD and associated memory loss, cognitive dysfunction, and neuronal death [18]. Similarly, high serum phosphate levels are associated with an increased incidence of dementia [23] and AD in humans [24,25], thus suggesting that phosphate overload is a high-risk factor for AD development. Together, these studies highlight the importance of calcium and phosphate homeostasis in preventing AD development and suggest that the dysregulation of calcium and phosphate homeostasis is a strong risk factor and key pathological event in AD development. Furthermore, elevated levels of parathyroid hormone (PTH), which is secreted in response to low serum calcium levels and has an essential role in maintaining phosphate homeostasis by influencing renal 1,25-dihydroxy vitamin D synthesis [26], has been associated with impaired cognitive function [27,28], dementia [29,30], and AD [31,32]. Although the involvement of PTH in these conditions remains controversial, elevated PTH may initiate or exacerbate AD pathogenesis in certain situations. For example, parathyroidectomy in patients with hyperparathyroidism has been shown to improve cognition [28,32]. These observations suggest that elevated PTH levels are associated with an increased risk of cognitive decline and AD incidence. However, how high levels of PTH stimulate the overexpression of APP and APP processing enzymes remains unclear.

Here, we found that rats fed diets with high-phytate and low-Ca^2+^ contents showed elevated expression of APP and APP processing enzymes in the hippocampus, thereby increasing Aβ and apoptotic neuronal cell death. Specifically, the expression of APP and BACE1 were found to be mediated by transcriptional activation through the direct binding of RXR and VDR to their promoters in response to elevated PTH. Thus, our studies provide novel insights into insoluble Aβ formation through the phytate-mediated dysregulation of calcium and phosphate.

## 2. Materials and Methods

### 2.1. Rats

Animal studies were approved by the Center for Animal Care and Use and were performed according to the institutional ethics and safety guidelines (LCDI-2008-0019, LCDI-2010-0068, and LCDI-2018-0060). Four-week-old female (40–45 g) Sprague Dawley rats (Orient Bio, Seoul, Korea) were housed at three animals per cage in a 12:12 h light–dark cycle with ambient temperature maintained at 22 ± 2 °C. The rats were fed AIN-93G diet (Dyets Inc., Bethlehem, PA, USA) for a 1-week adaptation period and were randomly assigned to the control AIN-93G (0% phytate) diet or AIN-93G with either 3% or 5% sodium phytate (P-8810, Sigma-Aldrich, St. Louis, MO, USA) for 18 weeks. All diets had equivalent percentages of carbohydrates, proteins, fats, and minerals. Pathogen-free water and food were available ad libitum. Food intake and body weight were recorded weekly. After 18 weeks of high-phytate diets, the animals were sacrificed to collect tissue samples for further analysis. 

### 2.2. Immunohistochemistry (IHC)

Formalin-fixed brain tissues from the rats after feeding them a high-phytate diet for 18 weeks were dehydrated with ethanol, embedded in paraffin, and sectioned at 3 µm. Antigen retrieval was performed in Tris-EDTA, at pH 9.0 for 3 min. For Aβ staining, the brain sections were further incubated in 70% formic acid for 15 min [11]. IHC was performed with antibodies on the following proteins at 25 °C for 2 h: Aβ (4G8, 1:200; Covance, US), BACE1 (61-3E7, 1:200; Abcam), PSEN1 (N-19, 1:200; Santa Cruz, Dallas, TX, USA), and cleaved caspase 3 (D175, 1: 200 Cell Signaling Technology, MA, USA). Horseradish peroxidase-conjugated secondary antibodies were detected with 3,3,-diamino-benzidine (DAB) tetrahydrochloride substrate (Dako, Glostrup, Denmark) or alkaline phosphatase (AP, GBI Labs, WA, USA). All sections were counterstained with hematoxylin (Thermo Scientific, MA, USA). Images were captured with a 3DHISTECH microscope (Budapest, Hungary).

### 2.3. Biochemical Assays

The rats were fasted overnight every 3–4 weeks and anesthetized with isoflurane. Tail vein blood samples were stored at 4 °C for 10–20 min, and serum was stored at −80 °C. Serum Ca^2+^ analysis was performed with an AU480 chemistry analyzer (Model AU-480; Beckman Coulter, Fullerton, CA, USA). Intact rat PTH was measured with enzyme-linked immunosorbent assays (Immutopics, San Clemente, CA, USA).

### 2.4. Image Quantification 

Images were taken with a 3DHISTECH digital microscope (Budapest, Hungary) under a 25× objective lens. Six independent images were collected for each group and used to count the positively stained areas of DAB, which forms brown precipitation at the location of horseradish peroxidase through catalyzation of Aβ, BACE1, and PSEN1, or alkaline phosphatase (AP), which is visualized through Fast Red substrate precipitation for cleaved caspase-3 positive cells with the software Fiji [33]. The graphs shown in the figures illustrate the percentages of the areas calculated.

### 2.5. qRT-PCR

Total RNA was extracted from whole-brain or hippocampal lysates with TRIzol reagent (Invitrogen, Carlsbad, CA, USA). cDNA synthesis and quantitative RT-PCR were performed with TB Green Premix Ex Taq cDNA Synthesis kit (Takara Bio, Tokyo, Japan) with 1 μg total of RNA plus random hexamers and a Bio-Rad CFX384 instrument (Bio-Rad, USA). *Cyclophilin A* was used as an internal control for normalization of expression values. The primer sequences used for qRT-PCR are shown in Appendix A Table A1.

### 2.6. Western Blotting 

Total protein was extracted from 15 mg of whole-brain or hippocampus samples with protein 1× lysis buffer (2× lysis buffer, 100 mM of Na_3_VO_4_, and 0.5 M of NaF) with 1× protease inhibitor (Roche, switzerland). A total of 20 μg of protein samples was loaded on 10% or 15% SDS-PAGE gels (Bio-Rad). The loaded samples were then transferred to a PVDF membrane (Millipore, Billerica, MA, USA) in transfer buffer (Tris, pH 8.3, glycine, and 10% methanol) at 4 °C for 2 h. The transferred membrane was blocked with 5% skim milk (Merck) for 30 min at room temperature and incubated with specific primary antibodies overnight at 4 °C in TSBS with 1% skim milk. The primary antibodies of the following proteins were used for Western blotting: APP (NAB228, 1:500; Santa Cruz), ADAM10 (T-17, 1:500; Santa Cruz), BACE1 (61-3E7, 1:1000; Abcam), PSEN1 (N-19, 1:500; Santa Cruz), VDR (D-6, 1:500; Santa Cruz), RXRα (D-20, 1:1000; Santa Cruz), RXRβ (L-20, 1:1000; Santa Cruz), and β-actin (AC-15, 1:1000; Santa Cruz). After secondary antibody incubation for 30 min at room temperature, the membrane was developed with an LAS 4000 imaging system (Fujifilm, Japan). The quantification of the protein expression was calculated with the software Fiji [33].

### 2.7. Transcription Assays

Mouse *vdr*, *rxrα*, and *rxrβ* sequences were cloned using the pcDNA6/V5-HisA vector, and mouse genomic DNA sequences for *app*, *bace1*, *psen1*, and the human genomic *bace1* sequences were cloned using the firefly luciferase reporter vector pGL4.14 (Promega, USA) or pGL3 with *pfu* DNA polymerase (Solgent, South Korea). Site-specific mutagenesis and deletion mutants were performed with a QuikChange Site-Directed Mutagenesis Kit (Stratagene, USA) with the gene-specific primers listed in Appendix B Table A2. All sequences were verified. HEK293 cells in 12-well plates were transfected with FuGENE (1–4 μL per 0.5 μg DNA; Roche), and a total of 1.0 μg of DNA (1.0 μg of pGL4.14/pGL3 luciferase reporter, 0.5–1.0 μg of wild-type or mutated pcDNA6/V5-HisA-VDR, pcDNA6/V5-HisA-RXRα, or pcDNA6/V5-HisA-RXRβ, 0.1 μg of pRL-TK-expressing control *Renilla* luciferase, and the remaining pcDNA6/V5-HisA). HX531 (SML2170, Sigma-Aldrich), a synthetic RXR antagonist, was dissolved in DMSO and added at 2.5, 5.5, and 10.0 μM. Cells were lysed 24–36 h after transfection, and the lysates were rocked at room temperature for 15 min and centrifuged at 8000 rpm for 10 min. A 30 μL volume of supernatant was subjected to luciferase assays with a Centro XS3 LB luminometer (Berhold, Germany). The ratios (firefly/*Renilla*) of relative luciferase activity were determined. 

### 2.8. Chromatin Immunoprecipitation Analysis

ChIP analysis was performed according to the manufacturer’s instructions with a ChIP-IT Express Kit (Active Motif, Carlsbad, CA, USA). Briefly, HEK293 cells at 80% confluency were harvested, washed with PBS, crosslinked with 1% formaldehyde, and sheared by sonication to fragments less than 200 bp. The sheared chromatin was immunoprecipitated with antibodies to total VDR (C-20, Santa Cruz, Dallas, TX, USA), RXRα (D-20, Santa Cruz, Dallas, TX, USA), or RXRβ (L-20, Santa Cruz, Dallas, TX, USA), or with IgG (ab6721, Abcam). DNA crosslinking was reversed overnight, and PCR was then performed with primer pairs recognizing three different regions of the human *bace1* promoter listed in Appendix C Table A3. The primer pair for *bace1* amplified a region containing multiple VDR-binding sites (−341/−453 and −1471/−1583) and RXR-binding sites (−261/−363 and −431/−553). PCR products were visualized after electrophoresis on a 2% agarose gel.

### 2.9. Statistical Analysis

Sample sizes for all experiments were based on preliminary results and previous experience in conducting related experiments. Power calculations were not used to determine sample sizes. Animals for each group of experiments were randomly assigned. Unless noted otherwise, all data are presented as the mean ± standard error of the mean (SEM). Statistical comparisons of groups were performed by either one-way ANOVA with Dunnett’s multiple comparison test or two-way ANOVA with Sidak’s multiple comparison test in the software GraphPad Prism 9 (GraphPad Software Inc., San Diego, CA, USA).

## 3. Results

### 3.1. Rats Fed High-Phytate Diets Show Elevated Accumulation of Aβ Peptide and Neuronal Apoptosis in the Hippocampus

**Figure 1:**
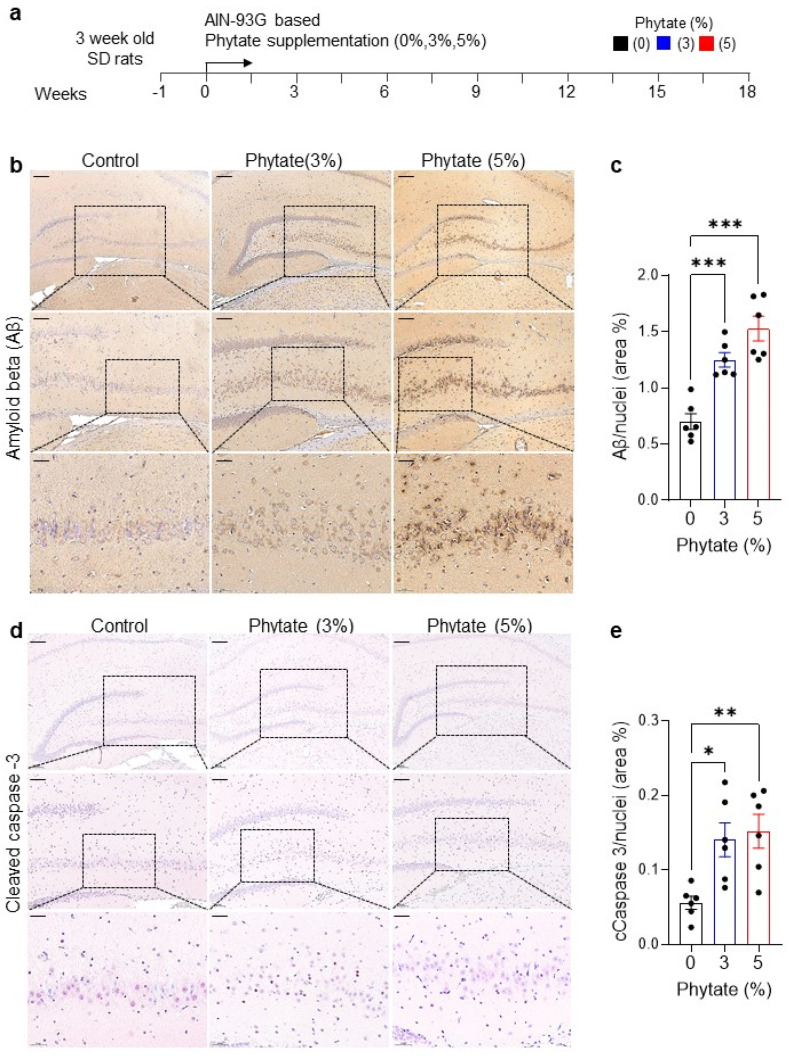
Rats fed a high-phytate, low-Ca^2+^ diet show elevated Aβ accumulation and apoptotic neuronal cell death in the hippocampus: (**a**) Experimental design. After a 1-week period of adaptation to the control AIN93G diet, 4-week-old female Sprague Dawley rats were fed AIN-93G diets with 0% (control), 3%, or 5% phytate supplementation for 18 weeks. (**b**) Representative immunohistochemistry images of Aβ staining in the brain sections in rats fed 0% (control), 3%, or 5% phytate diets after 18 weeks. Extracellular Aβ staining showed a distinctive distribution pattern of Aβ in the hippocampal CA3 and DG regions. Scale bars, 200 μm (low magnification), 100 μm (high magnification), and 50 μm (highest magnification). (**c**) Aβ positive cells in the hippocampal CA3 and DG regions were quantified in five to eight low-power fields (mean ± SEM, *n* = 5–10). (**d**) Representative immunohistochemistry images of cleaved caspase-3 staining in the brain sections in rats fed 0% (control), 3%, or 5% phytate diets after 18 weeks. Scale bars, 200 μm (low magnification), 100 μm (high magnification), and 50 μm (highest magnification). (**e**) Cleaved caspase-3 cells in the hippocampal CA3 and DG regions were quantified within five to eight low-power fields (mean ± SEM, *n* = 5–10). One-way ANOVA was used for all comparisons with Dunnett’s multiple comparison test. All data are presented as mean ± SEM for each group (*n* = 3–4 lpf/rat). * *p* < 0.05, ** *p* < 0.01, *** *p* < 0.001 compared with controls.

To examine the effects of high-phytate, low-calcium diets on Aβ formation, we investigated whether the high-phytate-mediated dyshomeostasis of calcium and phosphate might be sufficient to increase insoluble Aβ production. To address this question, we fed 4-week-old female Sprague Dawley rats AIN-93G diets with 0% (control), 3%, or 5% phytate supplementation for 18 weeks (Figure 1a). We performed immunohistochemical (IHC) staining of Aβ to examine Aβ accumulation in the hippocampus in rats. IHC analysis clearly showed significantly greater Aβ accumulation as neuritic plaques in the hippocampal cornu ammonis 3 (CA3) and dentate gyrus (DG) regions in rats fed high-phytate diets rather than the control diet (Figure 1b), thus indicating that the Aβ mainly accumulated in the peripheral regions of neuronal cells in rats fed high-phytate diets. The quantification of DAB signals showed that the Aβ deposition was almost three-fold higher in the hippocampal CA region in high-phytate-diet-fed rats than control rats (Figure 1c). Thus, these results suggest that the high-phytate diets, compared with the control diet, increased the formation of insoluble Aβ peptides in the hippocampus in rats.

Because Aβ accumulation in the brain is thought to be an early toxic event in the pathogenesis of AD [34,35,36,37] and Aβ accumulation in the brain potentiates neuronal cell death through reactive oxygen species-mediated apoptosis [38], we evaluated whether Aβ accumulation in the brain in rats fed high-phytate diets might be associated with neuronal cell death. To test this possibility, we performed IHC staining of cleaved caspase 3, a marker of programmed cell death [37,39], in the hippocampus in rats fed high-phytate diets or a control diet. IHC analysis clearly showed that the cleaved caspase 3-positive neuronal cells were significantly more abundant in the hippocampus in rats fed high-phytate diets rather than the control diet (Figure 1d). Furthermore, the quantification of cleaved caspase 3-positive cells showed that neuronal cell apoptosis increased in a phytate-dose-dependent manner (Figure 1e), thus demonstrating that rats fed high-phytate diets showed highly increased neuronal cell apoptosis in the hippocampus. Thus, these data suggest that the high-phytate diets increased Aβ accumulation, which may in turn have induced neuronal cell apoptosis in the rat hippocampus.

### 3.2. High-Phytate Diets Increase Aβ Accumulation through Activating the Amyloidogenic Pathway in the Hippocampus

**Figure 2:**
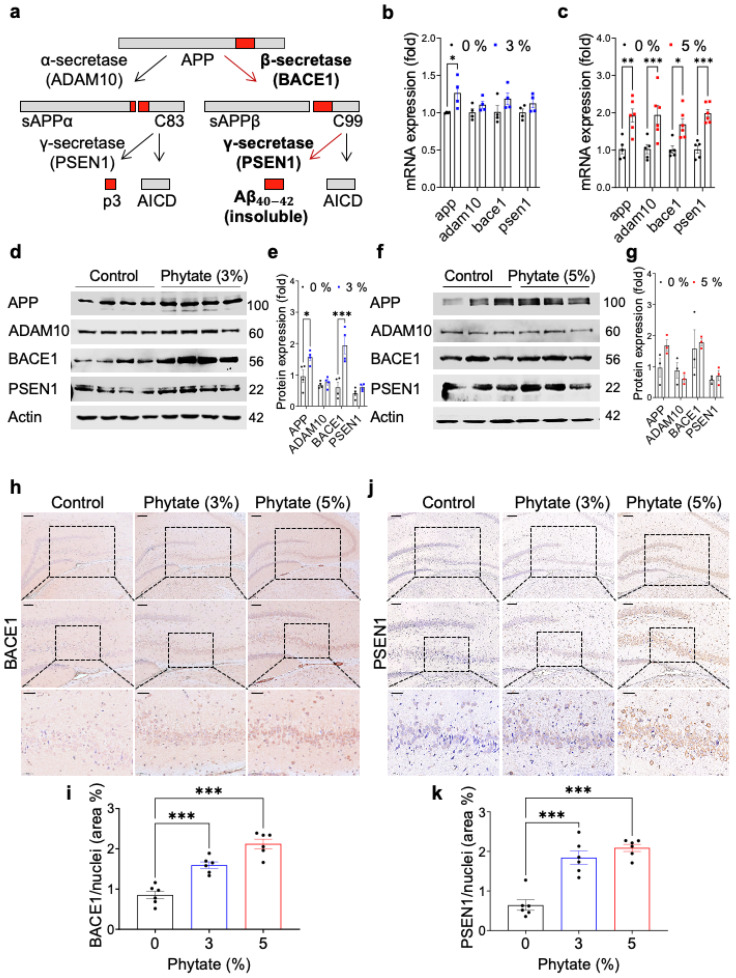
Rats fed high-phytate diets show elevated expression of APP and the BACE1 and PSEN1 amyloidogenic enzymes and increased Aβ accumulation in the hippocampus: (**a**) Schematic diagram of either amyloidogenic or non-amyloidogenic pathways. (**b**,**c**) The mRNA expression of *app*, *adam10*, *bace1*, and *psen1* in the whole brain in rats fed 0% (control, *n* = 4–6), 3% (*n* = 4) (**b**), or 5% (*n* = 6) (**c**) phytate diets after 18 weeks. (**d**,**e**) The protein expression of APP, ADAM10, BACE1, and PSEN in the whole brain in rats fed 0%, 3% (**d**,**e**), or 5% (**f**,**g**) phytate diets after 18 weeks. The molecular weight is marked to the right of the bands. The protein expression was quantified by calculation of the intensity of each band from the same blot and normalization to actin. (**h**) Representative immunohistochemistry images of BACE1 in the hippocampus in rats fed 0% (control, *n* = 4–6), 3% (*n* = 4), or 5% (*n* = 6) phytate diets after 18 weeks. Scale bars, 200 μm (low magnification), 100 μm (high magnification), and 50 μm (highest magnification). (**i**) The BACE1-positive cells in the hippocampal CA3 and DG regions were quantified in five to eight low-power fields (mean ± SEM, *n* = 5–10). (**j**) Representative immunohistochemistry images of PSEN1 in the hippocampus in rats fed 0% (control, *n* = 4–6), 3% (*n* = 4), or 5% (*n* = 6) phytate diets after 18 weeks. Scale bars, 200 μm (low magnification), 100 μm (high magnification), and 50 μm (highest magnification). (**k**). The PSEN1-positive cells in the hippocampal CA3 and DG regions were quantified in five to eight low-power fields (mean ± SEM, *n* = 5–10). IHC staining clearly showed that rats fed high-phytate diets showed more BACE1- or PSEN-positive cells in the hippocampal CA3 and DG regions. BACE1 and PSEN1 staining appeared as moss-like fiber sprouting in the hippocampal CA3 and DG regions. All data are presented as mean ± SEM for each group (*n* = 3–4 lpf/rat). * *p* < 0.05, ** *p* < 0.01, *** *p* < 0.001 compared with controls.

APP is best known as the precursor molecule that, after cleavage by β-secretase and γ-secretase (Figure 2a), produces 37–49 amino acid residue Aβ peptides, which are the primary component of the amyloid plaques found in the brains of people with AD [40]. To investigate how high-phytate diets increase the formation of insoluble Aβ peptides in the hippocampus in rats, we performed expression analysis of *app* and several genes involved in APP processing, such as *adam10*, *bace1*, and *psen1*, by qRT-PCR on whole-brain samples from rats fed 0%, 3%, or 5% phytate for 18 weeks. The mRNA levels of *app* were significantly higher in the whole brain in the phytate-fed groups than the control group (Figure 2b,c). In contrast, the mRNA levels of *adam10, bace1*, and *psen1*—which encode an α, β-, and γ-secretase, respectively—increased only in the rats fed 5% phytate (Figure 2c). These results suggest that rats fed high-phytate diets show increased expression of *bace1* and *psen1,* which are critical genes for forming insoluble Aβ peptides [41,42,43]. In agreement with the gene expression results, immunoblotting analysis of whole-brain lysates further confirmed that rats fed high-phytate diets showed significantly elevated protein levels of APP and BACE1 but not ADAM10 and PSEN1 (Figure 2d–g), thus suggesting that greater formation of Aβ peptides was strongly associated with the elevated levels of APP and APP processing enzymes such as BACE1 and PSEN1.

To further verify the expression of APP processing enzymes correlated with the regions of Aβ formation in the brain, we performed IHC staining for BACE1 and PSEN1. However, the IHC analyses showed increased expression of BACE1 and PSEN1 in a dose-dependent manner in the CA3 regions of the hippocampus (Figure 2h–k), where Aβ deposition occurs. Thus, these results suggest that the increased Aβ deposition in rats fed high-phytate diets might have resulted from the overexpression of BACE1 and PSEN1, which are critical enzymes for the formation of Aβ.

### 3.3. Rats Fed High-Phytate Diets Develop Secondary Hyperparathyroidism and Show Effects of PTH on the Transcriptional Activity of App and APP Processing Enzymes

**Figure 3:**
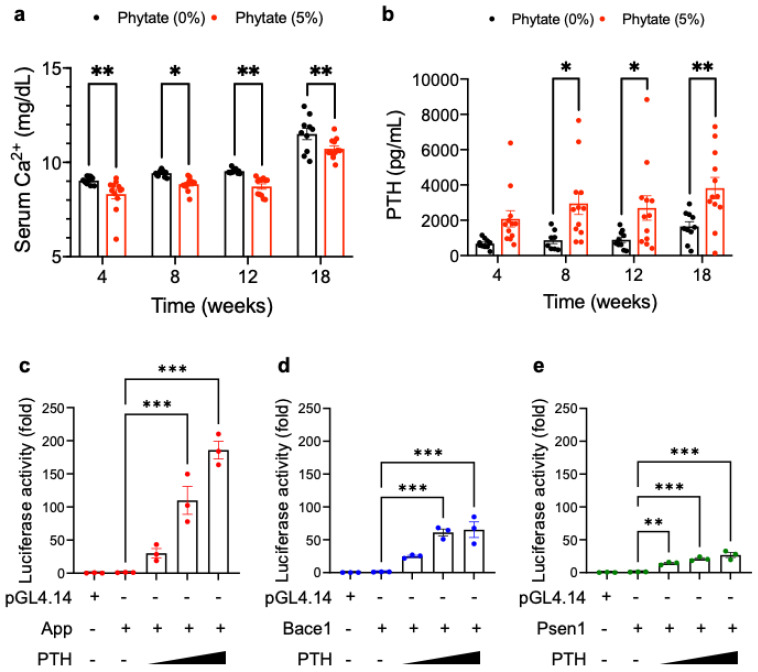
Rats fed high-phytate diets develop secondary hyperparathyroidism with hypocalcemia, and PTH affects the promoter activity of *app* and APP processing enzyme genes: (**a**) Serum levels of Ca^2+^ of all groups during 18 weeks of feeding with the experimental diets. (**b**). Serum levels of intact PTH were measured with enzyme-linked immunosorbent assay (ELISA). Two-way ANOVA was used for all comparisons with Sidak’s multiple comparison test. All data are presented as mean ± SEM for each group (*n* = 9–12). * *p* < 0.05, ** *p* < 0.01, *** *p* < 0.001 compared with controls. (**c**–**e**). The effects of PTH on the transcriptional activation of *app* (**c**), *bace1* (**d**), or *psen1* (**e**) mouse promoters. Standard luciferase-based transcriptional reporter assays were conducted in HEK293T cells transiently cotransfected with 0.1, 0.5, or 1.0 μg pcDNA-PTH; the luciferase vectors pGL4.14-*app*, pGL4.14-*bace1*, or pGL4.14-*psen1*; and 0.1 μg of pRL-TK *Renilla* luciferase to normalize for transfection efficiency. Data show the mean ratios for multiple experiments (±SEM). One-way ANOVA was used for all comparisons with Dunnett’s multiple comparison test. All data are presented as mean ± SEM for each group (*n* = 3–4 lpf/rat). * *p* < 0.05, ** *p* < 0.01, *** *p* < 0.001 compared with controls.

In agreement with findings from previous studies [11], we observed that high-phytate-fed rats developed hypocalcemia during all diet periods (Figure 3a). The serum levels of PTH had markedly increased in the 5% phytate-fed groups by 8 weeks and remained elevated until week 18 (Figure 3b). These results suggest that the rats fed high-phytate diets developed secondary hyperparathyroidism in response to hypocalcemia. Many diseases with elevated PTH, such as primary hyperparathyroidism and chronic kidney disease with secondary hyperparathyroidism, are associated not only with mineral bone disease but also an increased risk of impaired cognitive function and AD [27,30,31]. Therefore, to examine whether the high levels of PTH induced by phytate might affect the transcriptional activity at the *app*, *bace1*, and *psen1* gene promoters, we cloned the promoters of the *app*, *bace1*, and *psen1* mouse genes using the firefly luciferase reporter vector pGL14.4. 

Levels of reporter protein were assessed in HEK293 cells transiently cotransfected with PTH-expression vector and *app*-, *bace1*-, or *psen1*-promoter vectors. *Renilla* luciferase plasmids were cotransfected to normalize transfection efficiency. As expected, the absence of PTH had no significant effects on the regulation of *app*, *bace1*, or *psen1* promoter activity (Figure 3c–e). However, the overexpression of PTH resulted in a 30- to 180-fold enhancement in the transcriptional activity of APP reporter gene transcription over that of the basal vector pGL4.14 in luciferase reporter assays, depending on the amount of pcDNA (PTH) (Figure 3c). Similarly, transfection with PTH stimulated the transcriptional activity of both *bace1*- and *psen1*-reporter gene transcription by 10- to 70-fold that of the basal vector control in a dose-dependent manner (Figure 3d,e). Thus, these results suggest that the promoters of *app* and *bace1*, but not *psen1*, are highly responsive to PTH, thus leading to increased transcriptional activity. Therefore, we propose that under physiological conditions, high PTH contributes to upregulating the expression of APP and BACE1, thereby leading to Aβ deposition in the hippocampal CA region in rats fed high-phytate diets.

### 3.4. High-Phytate Diets Increase the Expression of APP and BACE1 through Transcriptional Activation by RXRα/β and VDR

**Figure 4:**
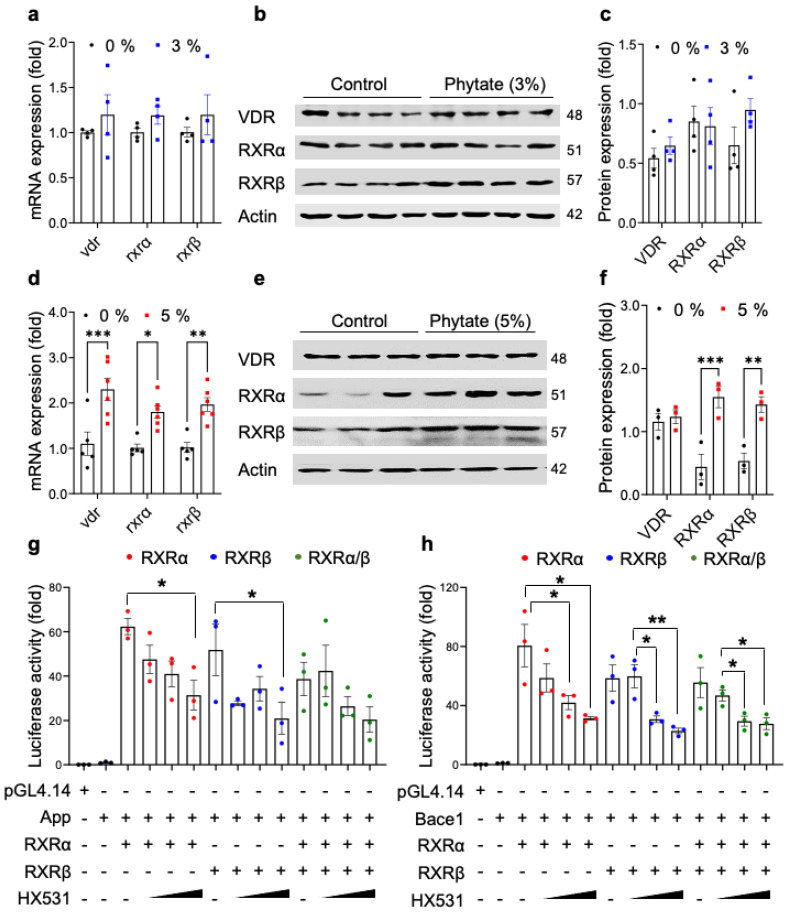
The RXR antagonist HX531 suppresses the transcriptional activity of *rxrα/β* and *vdr* in the *app* and *bace1* promoters in vitro: (**a**) The mRNA expression of *rxr*α, *rxr*β, and *vdr* in the whole brain in rats fed 0% (control, *n* = 4) and 3% (*n* = 4) phytate after 18 weeks of the diet. (**b**) Immunoblot analysis of RXRα, RXRβ, and VDR from the whole-brain lysates from rats fed 0% (control, *n* = 4) and 3% (*n* = 4) phytate after 18 weeks of the diet. The molecular weight is marked to the right of the bands. (**c**) The protein expression was quantified by calculation of the intensity of each band from the same blot and normalization to actin. (**d**) The mRNA expression of *rxr*α, *rxr*β, and *vdr* in the whole brain in rats fed 0% (control, *n* = 6) and 5% (*n* = 6) phytate after 18 weeks of the diet. (**e**) Immunoblot analysis of RXRα, RXRβ, and VDR from the whole brain lysates from rats fed 0% (control, *n* = 3) and 5% (*n* = 3) phytate after 18 weeks of the diet. The molecular weight is marked to the right of the bands. (**f**). The protein expression was quantified by calculation of the intensity of each band from the same blot and normalization to actin. (**g**,**h**) The effects of HX531, a synthetic RXR antagonist (2.5, 5.0, and 10.0 μM), on the transcriptional activity of the *app* (**g**) or *bace1* (**h**) mouse promoter. Standard luciferase–transcriptional reporter assays were conducted in HEK293T cells transiently cotransfected with RXRα, RXRβ, or RXRα/β expression plasmids; luciferase pGL4.14-*app* or pGL4.14-*bace1* vectors; and 0.1 μg of pRL-TK *Renilla* luciferase to normalize for transfection efficiency. At 24 h after the transfections, HEK293T cells were dose-dependently treated with HX531 for 6 h. Data show the mean ratios for multiple experiments (±SEM). One-way ANOVA was used for all comparisons with Dunnett’s comparison test. All data are presented as mean ± SEM for each group (*n* = 3–4 lpf/rat). * *p* < 0.05, ** *p* < 0.01, *** *p* < 0.001 compared with controls.

To identify potential transcription factors involved in the transcriptional activation of *app* and *bace1* in response to high PTH, we initially mapped the responsive regions on the promoters of the *app* and *bace1* genes by using GeneHancer [44]. The prediction results suggested that the retinoic acid receptor (RXRα/β) and vitamin D receptor (VDR) are the potential transcription factors binding the promoters of *app* and *bace1*. To further verify whether the expression of RXRα, RXRβ, and VDR was upregulated in the brain tissues in rats fed high-phytate diets, we performed qRT-PCR and immunoblotting analyses on brain tissues. Rats fed 3% phytate, compared with the control diet, did not show altered expression of RXRα and RXRβ in the whole brain (Figure 4a–c). In agreement with the GeneHancer predictions and mapping results, rats fed 5% phytate, compared with the control diet, showed elevated expression of both RXRα and RXRβ in the whole brain, at both the mRNA and protein levels (Figure 4d–f). In contrast, the expression of VDR did not change at the protein levels with 5% phytate diet feeding (Figure 4d–f). These results suggested that rats fed 5% phytate showed elevated expression of APP and the APP processing enzyme BACE1, in a manner potentially driven by transcriptional activation by the RXR/VDR heterodimeric complex in the hippocampus.

To further investigate whether ligand inhibition of RXR might affect the transcriptional activity of *app1* and *bace1* promoters, we transiently coexpressed RXRα, RXRβ, or RXRα/β-expression plasmid together with the *app* or *bace1* mouse promoter vector in HEK293 cells to assess transcriptional activity. We subsequently treated the cells with HX531, a synthetic RXR antagonist, which inhibits the activation of both RXR homodimers and RXR heterodimers [45]. The expression of RXRα, RXRβ, or both RXRα/RXRβ markedly increased the transcriptional activity of the *app* and *bace1* promoters (Figure 4g,h). In contrast, treatment with HX531 markedly suppressed the RXRα/β-stimulated transcriptional activity of the *app* and *bace1* promoters (Figure 4g,h), thus indicating that both RXRα and RXRβ play essential roles in the transcriptional activation of both the *app* and *bace1* genes. These experiments suggested that rats fed high-phytate diets showed elevated expression of *app* and *bace1* through transcriptional activation by RXRα/β.

### 3.5. The Transcriptional Activation of App and bace1 Is Mediated by Direct Promoter Binding of RXR and VDR

**Figure 5:**
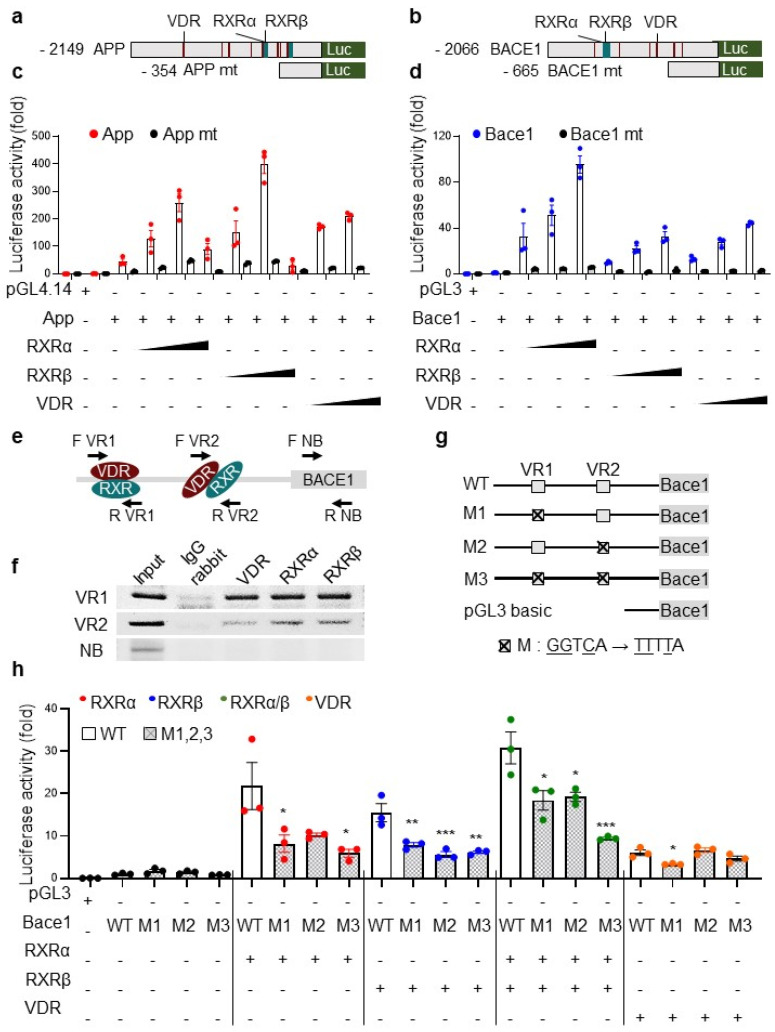
The transcriptional activation of app and bace1 is mediated by the direct promoter binding of RXRα/β and VDR: (**a**,**b**) Diagrams of *app* (**a**) and *bace1* (**b**) mouse promoters containing RXR and VDR binding sites, or deleted RXR and VDR binding sites: *app* mt (**a**) or *bace1* mt. (**b**) The transcriptional binding sites of RXR and VDR were predicted by the programs TRANSFAC and Vector NTI. (**c**–**d**) The effects of transcriptional activation of *rxrα*, *rxrβ*, or *vdr* on various mouse truncated luciferase reporters of pGL4.14-*app* (**c**) or pGL3-*bace1*. (**d**) RXRα, RXRβ, or VDR was transiently coexpressed with various truncated pGL4.14-*app* or pGL3-*bace1* luciferase reporters, and pRL-TK *Renilla* luciferase in HEK293T cells for 24 h. Data show the mean ratios for multiple experiments (±SEM). (**e**) A schematic diagram of RXR or VDR responsive element binding sites on the human *bace1* promoter region with the detection primer sets for ChIP in Figure 5f: VR1 (VDR/RXR binding region 1), VR2 (VDR/RXR binding region 2), and NB (non-binding region). F and R denote the forward and reverse primer, respectively, and the detection primer regions are shown by arrows. (**f**) After chromatin from HEK293 cells was immunoprecipitated with IgG or antibodies to RXRα, RXRβ, or VDR, the precipitated chromatin was subjected to PCR with primers specific to three regions (VR1, VR2, and NB) of the *bace1* promoter, as indicated in (**e**). (**g**) Schematic diagram of various mutations affecting the binding sites of RXR or VDR in the human *bace1* promoters. (**h**) The effects of transcriptional activation of RXRα, RXRβ, or VDR on the binding site mutants in the pGL4.14-*bace1* luciferase reporter. RXRα, RXRβ, or VDR was transiently coexpressed with various binding site mutants in the pGL4.14-*bace1* luciferase reporter, and pRL-TK *Renilla* luciferase in HEK293T cells for 24 h. Data show the mean ratios for multiple experiments (±SEM). One-way ANOVA was used for all comparisons with Dunnett’s multiple comparison test. All data are presented as mean ± SEM for each group (*n* = 3–4). * *p* < 0.05, ** *p* < 0.01, *** *p* < 0.001 compared with controls.

As expected, given that the RXR and VDR heterodimer complex binds vitamin D responsive elements (VDREs) in gene promoters [12] under the control of PTH [13], our mapping studies showed that the promoters of *app* and *bace1* contain multiple VDREs (Figure 5a,b). To verify putative binding sites of RXRα/RXRβ and VDR, we generated deletion constructs of *app* and *bace1* mouse promoters lacking the putative binding sites for RXRα/RXRβ and VDR (Figure 5a,b). We then examined the transcriptional activity after transient cotransfection with wild-type (WT) or deletion mutants for *app* and *bace1* together with vectors for expression of RXRα, RXRβ, or VDR in HEK293 cells. After normalization, transfection with RXRα, RXRβ, or VDR dose-dependently stimulated the transcriptional activity of WT *app* and WT *bace1* reporter gene transcription by ~10- to 400-fold that in mock-transfected cells (Figure 5c,d). Notably, the deletion mutant constructs lacking the responsive element abolished the transcriptional activity of the *app* or *bace1* promoter via RXRα, RXRβ, or VDR (Figure 5c,d), thus suggesting that the transcriptional activity of *app* or *bace1* might be regulated by the RXR/VDR heterodimer complex. Again, these results suggested that the RXR/VDR heterodimer plays an essential role in the transcriptional activation of *bace1*.

BACE1, the essential and exclusive APP-cleaving enzyme generating Aβ [3,41,42], and increased BACE1 in AD brains, plays a critical role in AD pathophysiology [46], thus implying that BACE1 might be an important drug target for slowing Aβ production in early AD. According to our findings that RXRα/β and VDR directly increased the transcriptional activity of the *bace1* promoter, we further examined whether RXRα/β and VDR might directly bind *bace1* DNA by performing chromatin immunoprecipitation (ChIP) analysis. After the DNA from HEK293 cells was cross-linked and mechanically sheared, the chromatin was then immunoprecipitated with IgG or antibodies to RXRα, RXRβ, or VDR, and then PCR was performed with primers amplifying three regions: VDR/RXR binding region 1 (VR1), VDR/RXR binding region 2 (VR2), and a non-binding region (NB) of the *bace1* promoter (Figure 5e and Appendix C Table A3). Samples that were incubated with IgG antibody as a negative control did not generate PCR products. However, the VR1 primer set showed almost four-fold higher amplification levels than the VR2 primer set (Figure 5f), thereby confirming that the VDR and RXR heterodimer binds the VR1 and VR2 regions of the *bace1* promoter. These results indicate that both binding sites are targeted by RXR/VDR heterodimers.

Finally, we further confirmed the direct binding of RXRα/β and/or VDR to the promoter region of *bace1*. To do so, we generated three human mutant constructs (Figure 5g) that lacked the consensus binding sites of RXR/VDR (M1), RXR (M2), or both (M3). We transiently cotransfected the RXRα/RXRβ expression vector with a combination of pGL14.1-*bace1* luciferase reporters bearing WT, M1, M2, M3, or the empty vector in HEK293 cells. The overexpression of RXRα, RXRβ, RXRα/RXRβ, or VDR markedly increased the transcriptional activity of the WT *bace1* luciferase reporter. Interestingly, RXRα/RXRβ overexpression strongly stimulated the transcriptional activity (Figure 5h). However, the coexpression of all mutant constructs did not promote the transcriptional activity of the *bace1* promoter by overexpression of a combination of RXRα, RXRβ, RXRα/RXRβ, and VDR (Figure 5h). Notably, mutants lacking either of two consensus sites showed abolished *bace1* promoter transcriptional activity (Figure 5h). Thus, these results demonstrate that RXRα/β and VDR play essential roles in regulating the transcriptional activation of *bace1* through direct physical interaction with the promoter region. 

## 4. Discussion

We previously reported that a high-phytate, low-calcium diet is a crucial risk factor for phosphate overloading and renal phosphate wasting associated with crystal nephropathy, renal fibrosis, bone loss, and secondary hyperparathyroidism [11]. Here, we studied whether high-phytate diets might contribute to Aβ accumulation, a central pathological feature of AD. We found that rats fed high-phytate diets showed greater Aβ accumulation and an elevated rate of apoptotic neuronal cell death in the hippocampus. Thus, we propose that high-phytate diets are a substantial risk factors for increased Aβ accumulation and apoptotic neuronal cell death in this rat model.

IHC staining further indicated that rats fed high-phytate diets showed increased Aβ deposition, a hallmark of sporadic AD, in the C3A hippocampus region, where Aβ accumulates in patients with AD [47]. Furthermore, in agreement with the well-known properties of Aβ-induced neurotoxicity [48], the IHC staining of cleaved caspase 3 confirmed that rats fed high-phytate diets showed an elevated apoptotic neuronal cell death rate in the hippocampus. These results may explain why the accumulation of Aβ increased apoptotic neuronal cell death in the hippocampus in rats fed high-phytate diets. Our findings further show that elevated expression of both the β-secretase BACE1 and the γ-secretase PSEN1, both of which catalyze transmembrane cleavage of APP and generate Aβ [1], led to an increase in Aβ deposition in the C3A hippocampus in rats fed high-phytate diets. Our findings further support reports from previous studies indicating that the level of BACE1 protein in the presynaptic dystrophic neurites surrounding amyloid plaques increases the generation of Aβ plaques in a mouse model of AD [49] and in patients with AD [50]. Thus, our studies further support that overexpression of BACE1 in rats fed high-phytate diets might increase the cleavage of APP and Aβ deposition in the C3A hippocampus.

Previously, we demonstrated that high-phytate and low-calcium diets can cause dual negative effects in rats [11] by negatively affecting calcium and phosphate homeostasis through inhibiting calcium absorption while also aggravating intestinal phosphate overload, which eventually leads to high levels of PTH and active 1,25(OH)_2_D. Thus, these findings highlight the effects of a high-phytate diet on raising serum levels of PTH secondarily to calcium and phosphate dysregulation. In addition, recent clinical studies have shown that elevated PTH levels are associated with an increased risk of cognitive decline and incident dementia in a general older population [27], independently of calcium and renal function. Interestingly, several studies of patients with increased levels of PTH after parathyroidectomy normalized PTH have indicated marked improvement in cognitive function and signs of dementia [51,52]. Thus, these studies suggest that higher levels of PTH are associated with an increased risk of cognitive decline and AD development.

We also found that APP and BACE1 are transcriptionally upregulated in response to PTH stimulation in HEK293 cells, thus supporting that the high levels of PTH in rats fed high-phytate diets contributed to upregulating APP and BACE1 expression. In agreement with the gene expression and immunoblot analyses indicating that rats fed high-phytate diets showed markedly increased levels of RXRα and RXRβ in the whole brain, promoter mapping analysis further supported that the responsive regions of *app* and *bace1* promoters contain numerous binding sites for RXRα/β and VDR—transcription factors known to be physiological downstream targets of PTH [53]. The pharmacological inhibition of RXR with HX531, a synthetic RXR antagonist that inhibits the activation of both RXR homodimers and RXR heterodimers [45], suppressed the transcriptional activity of *app* and *bace1* promoters in a dose-dependent manner. These data support the deletion analysis of the RXR and VDR binding sites on *app* and *bace1* promoters, thus indicating that RXR and VDR play critical roles in the transcriptional activation of the *app* and *bace1* promoters. Finally, the DNA ChIP of RXRα, RXRβ, and VDR directly confirmed that the RXR/VDR heterodimer or RXR homodimer binds the *bace1* promoter. ChIP analysis further indicated that the *bace1* binding-site mutant promoter activity was abolished in the presence of RXR or VDR overexpression. Thus, our studies strongly suggest that enhanced amyloidogenic processing in rats fed high-phytate diets might result from APP and BACE1 upregulation through transcriptional activation of the RXR/VDR hetero- or homodimer in rats’ responses to high-phytate diets.

We additionally assessed the effects of a synthetic RXR antagonist, HX531, which inhibits the activation of RXR [45], on the transcriptional activation of the *app* and *bace1* promoters. Treatment with the RXR antagonist HX531 markedly suppressed the RXRα/β-mediated transcriptional activity of the *app* and *bace1* promoters, thereby demonstrating that RXRα/β plays an essential role in regulating the transcriptional activity of *app* and *bace1*. Thus, these data support that the enhanced amyloidogenic processing in rats fed high-phytate diets might have resulted from transcriptional upregulation of both APP and BACE1 through the transcriptional activation of RXRα/β, thus eventually leading to amyloidogenic pathway activation and Aβ plaque formation in rats fed high-phytate diets. In contrast to our observations, previous studies have suggested that RXR agonists rapidly stimulate Aβ clearance, thereby improving cognitive and behavioral deficits in mouse models of AD[54]. Nevertheless, long-term treatment with RXR agonists has not been found to decrease Aβ plaque formation [55,56], thus suggesting a different role of RXR on Aβ plaques during long-term treatment. Furthermore, our in vitro ChIP experiments showed that RXRα, RXRβ, and VDR clearly directly bound the promoters of *app* and *bace1* in HEK293 cells. Although the expression of VDR in the whole brain in rats fed high-phytate diets did not increase, we previously showed that high-phytate diets increase the levels of active 1,25(OH)_2_D [11], which might stimulate VDR’s transcriptional activation of the *app* and *bace1* promoters. Thus, our findings suggest the crucial roles of RXR and VDR in the transcriptional activation of APP and BACE1, thereby eventually stimulating amyloidogenic pathway activation and Aβ plaque formation. However, further research is needed to address whether RXR antagonist treatment might diminish Aβ plaques.

In conclusion, we demonstrated that high-phytate diets in rats contribute to Aβ accumulation and increased apoptotic neuronal cell death in the hippocampus. Furthermore, high-phytate-mediated dysregulation of Ca^2+^ and phosphate may directly stimulate the transcriptional activation of the *app* and *bace1* promoters by RXR and VDR in the hippocampus. Thus, our studies provide novel insights into high-phytate-mediated dysregulation of Ca^2+^ and phosphate as a risk factor for Aβ accumulation and apoptotic neuronal cell death.

## Data Availability

Not applicable.

## Appendix A

**Table A1:** qRT-PCR primer information used for detecting mRNA expression levels in this study (Figure 2b,c and Figure 4a,d).

	Gene	GeneBank Accession No.	Primer	Primer Sequences
1	App	NM_019288.2	Forward	5′-GATTCCTTACCGGTGCCTAGTTG
			Reverse	5′-TCCTGGTGTAGAAACTTGCACTTG
2	Adam10	NM_019254.1	Forward	5′-GAGCTCAGCTGGTGGCTTACA
			Reverse	5′-AACGCAGAAATCAGACAATGACA
3	Bace1	NM_019204.2	Forward	5′-TCGTTTGCCCAAGAAAGTATTTG
			Reverse	5′-AAGCCATCCGGGAACTTCTC
4	Psen1	NM_019163.3	Forward	5′-CCTACTTCCAGAATGCACAGATGT
			Reverse	5′-GCTGCCGTTCTTGATTGTCA
5	RXRα	NM_012805.3	Forward	5′-AAACCCCCTCTAGGCCTCAA
			Reverse	5′-GATGTGCTTGGTGAAGGATGAC
6	RXRβ	NM_206849.3	Forward	5′-GCACAGAAACTCAGCCCATTC
			Reverse	5′-TCACGCATTTTGGACACTAGCT
7	VDR	NM_017058.2	Forward	5′-TCCCAGGATTCAGGGATCTCA
			Reverse	5′-TGAAAGACTGGTTGGAGCGT
8	Cyclopillin	NG_042086.1	Forward	5′-GCCATTCCTGGACCCAAAA
			Reverse	5′-GGTCTTTGGGAAGGTGAAAGAA

## Appendix B

**Table A2:** The cloning primer information used for luciferase reporter assays in this study (Figure 3c–e, Figure 4g,h and Figure 5a–d,g,h).

	Gene	GeneBank Accession No.	Genecode No.	Primer	Primer Sequences
1	App	NM_007471	ENSMUST00000005406.11	Forward	5′-AAAGGTACCTGTATGTACATGCCTATGTG
				Reverse	5′-AAAAGATCTCGTGATCCTGCGTGGGCC
2	Bace1	NM_011792	ENSMUST00000034591.10	Forward	5′-AAA GGT ACC CTG TCG GATCCCAGTGACT
				Reverse	5′-AAAAGATCTCAGGGACTCCGGGCTCCTA
3	Psen1	NM_001362271	ENSMUST00000041806.12	Forward	5′-AAAGAGCTCAAGAATCTATTGGAACAC
				Reverse	5′-AAAGATATCAAGGCTGCTCTCAGTCTG
4	App mt	NM_007471	ENSMUST00000005406.11	Forward	5′-AAGGTACCACTCCCCAACTTGGTTCC
				Reverse	5′-AAAAGATCTCGTGATCCTGCGTGGGCC
5	Bace1 mt	NM_011792	ENSMUST00000034591.10	Forward	5′-AAA GGT ACC ATG GGA GAA GACCCACTG
				Reverse	5′-AAAAGATCTAGGCCACCATAATCCAGC
6	Bace1 WT	NM_012104	ENST00000313005.10	Forward	5′-AAA GGT ACC ATG GGA GAA GAC CCA CTG
				Reverse	5′-AAA AGA TCT AGG CCA CCA TAA TCC AGC
7	Bace1 M1 (VR2)	NM_012104	ENST00000313005.10	Forward	5′-GCATGAGATTTTATTGAACCCGGGAGGCGGAGGTTGC
				Reverse	5′-TCCCGGGTTCAATAAAATCTCATGCCTCAGCCTCTGAGTA
8	Bace1 M2 (VR1)	NM_012104	ENST00000313005.10	Forward	5′-GGATCACGATTTTAGGAGATCGAGACCATCTTGGC
				Reverse	5′-TCGATCTCCTAAAATCGTGATCCTCCCGCCTCGGCCT

## Appendix C

**Table A3:** The ChIP assay primer information used for detecting binding sequences in this study (Figure 5e,f).

	Targeting Element Binding Sites	Detection Site	Primer	Primer Sequences
1	VDR/RXR 1 (VR1)	−1538	Forward	5′-ATGATCGGCCGGGCGCG
		−1379	Reverse	5′-GCCTGCAACCACGCCCCGCT
2	VDR/RXR 2 (VR2)	−541	Forward	5′-ACAAACAGGTTCAGATGG
		−414	Reverse	5′-TTTTCCAGGCTGCAAAC
3	Non binding (NB)	101	Forward	5′-CGGGAGCTGCGAGCCGCG
		230	Reverse	5′-GGCGGGCCGGTGGCGGC
